# Draft Genome Sequence of Streptomyces cavourensis YBQ59, an Endophytic Producer of Antibiotics Bafilomycin D, Nonactic Acid, Prelactone B, and 5,11-Epoxy-10-Cadinanol

**DOI:** 10.1128/MRA.01056-18

**Published:** 2018-09-20

**Authors:** Huy Quang Nguyen, Nguyen Thi-Hanh Vu, Ha Hoang Chu, Son Ky Chu, Ha Hoang, Thanh Trung Tran, Cuong Nguyen, Linh Thi-My Dinh, Hien Thi-Thu Trinh, Tien Quyet Phi

**Affiliations:** a University of Science and Technology of Hanoi (USTH), Vietnam Academy of Science and Technology (VAST), Hanoi, Vietnam; b Institute of Biotechnology (IBT), Vietnam Academy of Science and Technology (VAST), Hanoi, Vietnam; c Hanoi University of Science and Technology (HUST), Hanoi, Vietnam; d Gene Technology Department, Vinmec Research Institute of Stem Cell and Gene Technology, Hanoi, Vietnam; e Graduate University of Science and Technology (GUST), Vietnam Academy of Science and Technology (VAST), Hanoi, Vietnam; Queens College

## Abstract

This study reports the draft genome sequence of the endophytic Streptomyces cavourensis strain YBQ59, produces the antibiotics bafilomycin D, nonactic acid, prelactone B, and 5,11-epoxy-10-cadinanol. The draft genome sequence comprises ∼10.2 Mb, with a GC content of 64% and 8,958 predicted protein-coding genes, of which 14 gene clusters were found to associate with antibiotic biosynthetic pathways.

## ANNOUNCEMENT

The endophyte Streptomyces cavourensis strain YBQ59 was isolated from the root of the medicinal plant *Cinnamomum cassia* Presl in Vietnam (1). Eight pure secondary metabolites were purified from culture broth of the strain YBQ59, among which the five compounds 1-monolinolein, bafilomycin D, nonactic acid, daidzein, and 3′-hydroxydaidzein showed antimicrobial and cytotoxic activities against multidrug-resistant bacteria and human cancer cell lines, respectively (1).

The strain S. cavourensis YBQ59 was grown on YIM38 medium (1) for 48 h, and cells were harvested by centrifugation at 10,000 rpm for 5 min. Genomic DNA was extracted by using the PureLink genomic DNA kit (Invitrogen, CA, USA), and the purity was determined with a NanoDrop spectrophotometer (ThermoFisher Scientific, Inc., Waltham, MA, USA). The genomic DNA of the strain YBQ59 was sequenced by using Ion Torrent PGM technology with the Ion PGM template OT2 200 and sequencing 200 kits (ThermoFisher Scientific, Inc.). The obtained raw data consisted of 512,914,080 bp, comprising 2,856,000 reads (25 to 373 bp), in which the quality was controlled by using Trimmomatic (with parameters Slidingwindow, 10:30; Crop, 215; Minlen, 50) (2). The *de novo* genome assembly was performed by using VelvetOptimiser with default parameters (3), which revealed the genome size to be 10,232,294 bp, comprising 4,428 contigs (*N*_50_, 12,307 bp; longest contig length, 161,472 bp) with coverage of 26× and an average GC content of 64%. Gene prediction and annotation were processed by combining three programs, Prodigal (4), GeneMarkS (5), and NCBI Prokaryotic Genome Annotation Pipeline (PGAP) version 4.5 (6) with default parameters, which allowed for the identification of 8,958 overlapped predicted protein-coding sequences, 76 tRNA genes, 4 complete rRNA operons, and 3,145 pseudogenes. Blast2GO PRO (7) was used for the functional annotation of the whole genome based on Gene Ontology (GO) databases, which showed 6,292, 3,083, and 5,961 genes assigned to known biological processes, synthesis of cellular components, and molecular functions (GO categories), respectively. Analysis of the genome annotation based on the Kyoto Encyclopedia of Genes and Genomes (KEGG) database (8) revealed at least 140 distinct metabolic pathways. Notably, 717 enzymes linked to secondary metabolic biosynthesis were predicted, among which 158 enzymes are directly involved in antibiotic biosynthetic pathways, such as macrolides; ketolides; 12-, 14-, and 16-membered macrolides; nonribosomal peptide structures; and polyketide type I and type II structures. The biosynthetic pathways of the specific antibiotics cephamycin C, nocardicin A, clavaminate, erythromycin A/B, oleandomycin, picromycin, methymycin, neomethymycin, avermectin, tetracycline, oxytetracycline, chlortetracycline, mithramycin, tetracenomycin C, alamycin, rebeccamycin, vancomycin, and validamycin A were predicted based on the KEGG database (8), supporting the broad-spectrum antimicrobial activity of the strain YBQ59.

Furthermore, analysis using antiSMASH v3.0 (9) with default parameters revealed that the S. cavourensis YBQ59 genome contains 37 putative biosynthetic gene clusters (BGCs) involved in 14 different biosynthetic pathways, including the following: nonribosomal peptide synthetase (NRPS), type II polyketide synthases (PKS-II), hybrid PKS-NRPS, type III polyketide synthase (PKS-III), beta-ketoacyl synthase, and other BGCs for producing bacteriocin, bleomycin, calyculin, coelimycin, colonic acid, chlorizidine A, desferrioxamine B, ectoine, fatty acids, macrotetrolides, naringenin, landepoxcin, terpenes, and svaricin. Of those antibiotics, macroterrolides exhibiting the broad spectrum of antifungal, antibacterial, and anticancer properties have been found in Streptomyces griseus DSM 40695 (10). The analyzed genomic data provide valuable insights into the genetic determinants associated with antimicrobial and cytotoxic properties of S. cavourensis YBQ59 (1).

### Data availability.

The draft genome sequence of the strain Streptomyces cavourensis YBQ59 has been deposited at DDBJ/ENA/GenBank under the accession number QLNH00000000. The version described in this paper is the first version, QLNH01000000.
